# Report of Case of Twin Pregnancy with Distinct Placenta, One of Which Was a Placenta Prævia

**Published:** 1893-10

**Authors:** Fred V. Turk

**Affiliations:** Stilesboro, Ga.


### REPORT OF CASE OF TWIN PREGNANCY WITH DISTINCT PLACENTA, ONE OF WHICH WAS A PLACENTA PRÆVIA.

BY FRED V. TURK, STILESBORO, GA.

On the 26th ult. at 11 o'clock p. m., I was called to see Mrs. L., age 31, multipara, whom the messenger said was "about to be confined." Upon my arrival I found patient suffering but little, only "sharp cutting pains" as she described them; on examination I found considerable hemorrhage, and the os but slightly dilated, if at all, she thought she was not more than six or seven months in pregnancy. I prescribed for her and left, with instructions that I be called should any alarming symptoms appear.

Late in the afternoon of the 27th, I was hurriedly called, and upon my arrival found her suffering greatly, and upon examination found the os dilated to about the size of a silver dollar and a very profuse hemorrhage. I saw at once that premature labor was inevitable.

Upon my second examination I found the os more widely dilated, and introducing my fingers I felt a thick membrane of some kind protruding, completely enveloping the internal os. The hemorrhage was so great that it ran through the bedding and stood in a pool upon the floor. The patient was very weak, vomiting severely and frequently, she would faint so to speak, and appear almost lifeless. I saw at once something must be done, so introduced my whole hand into the vagina, and my fingers into the internal os and detached the thick membrane from the walls of the uterus all around, and then the second or third pain expelled the membrane, which proved to be a placenta and the following pain expelled the fœtus, (male) which was dead, and looked as though it had been carried to maturity, as regards size and development. The patient was still suffering greatly, and to my surprise, on examination, I found another fœtus with a foot presentation, the second fœtus was delivered, also dead. I severed the cord and extracted the placenta, which was attached to the fundus of the uterus. Now I had two fœtus, two distinct placenta and cords. After washing out the uterus thoroughly I left and returned in a few hours to find patient resting quietly, and to-day she is enjoying her usual good health.

This case no doubt, is of rare occurrence, I have neither read or heard of a case as this was.

In speaking of fœtus No. 2, I neglected to say that it was a female, and about half the size of the first one, and I am unable as yet to satisfy my mind as to what stage of pregnancy she was in at the time.

